# CX_3_CL1 (fractalkine) and CX_3_CR1 expression in myelin oligodendrocyte glycoprotein-induced experimental autoimmune encephalomyelitis: kinetics and cellular origin

**DOI:** 10.1186/1742-2094-2-17

**Published:** 2005-07-29

**Authors:** Dan Sunnemark, Sana Eltayeb, Maria Nilsson, Erik Wallström, Hans Lassmann, Tomas Olsson, Anna-Lena Berg, Anders Ericsson-Dahlstrand

**Affiliations:** 1 Department of Molecular Sciences, AstraZeneca R&D Södertälje, S-151 85 Södertälje, Sweden; 2 Neuroimmunology Unit, Department of Clinical Neuroscience, Karolinska Institutet, S-171 76 Stockholm, Sweden; 3 Neurological Institute, University of Vienna, Austria; 4 Safety Assessment, AstraZeneca R&D Södertälje, S-151 85, Södertälje, Sweden

**Keywords:** chemokines, fractalkine, neuron, astrocyte, microglia, neuroinflammation, multiple sclerosis

## Abstract

**Background:**

Multiple sclerosis (MS) is a chronic inflammatory disease of the central nervous system (CNS). It is associated with local activation of microglia and astroglia, infiltration of activated macrophages and T cells, active degradation of myelin and damage to axons and neurons. The proposed role for CX_3_CL1 (fractalkine) in the control of microglia activation and leukocyte infiltration places this chemokine and its receptor CX_3_CR1 in a potentially strategic position to control key aspects in the pathological events that are associated with development of brain lesions in MS. In this study, we examine this hypothesis by analyzing the distribution, kinetics, regulation and cellular origin of CX_3_CL1 and CX_3_CR1 mRNA expression in the CNS of rats with an experimentally induced MS-like disease, myelin oligodendrocyte glycoprotein (MOG)-induced autoimmune encephalomyelitis (EAE).

**Methods:**

The expression of CX_3_CL1 and its receptor CX_3_CR1 was studied with *in situ *hybridization histochemical detection of their mRNA with radio labeled cRNA probes in combination with immunohistochemical staining of phenotypic cell markers. Both healthy rat brains and brains from rats with MOG EAE were analyzed. In defined lesional stages of MOG EAE, the number of CX_3_CR1 mRNA-expressing cells and the intensity of the in situ hybridization signal were determined by image analysis. Data were statistically evaluated by ANOVA, followed by Tukey\primes multiple comparison test.

**Results:**

Expression of CX_3_CL1 mRNA was present within neuronal-like cells located throughout the neuraxis of the healthy rat. Expression of CX_3_CL1 remained unaltered in the CNS of rats with MOG-induced EAE, with the exception of an induced expression in astrocytes within inflammatory lesions. Notably, the brain vasculature of healthy and encephalitic animals did not exhibit signs of CX_3_CL1 mRNA expression. The receptor, CX_3_CR1, was expressed by microglial cells in all regions of the healthy brain. Induction of MOG-induced EAE was associated with a distinct accumulation of CX_3_CR1 mRNA expressing cells within the inflammatory brain lesions, the great majority of which stained positive for markers of the microglia-macrophage lineage. Analysis in time-staged brain lesions revealed elevated levels of CX_3_CR1 mRNA in microglia in the periplaque zone, as well as a dramatically enhanced accumulation of CX_3_CR1 expressing cells within the early-active, late-active and inactive, demyelinated lesions.

**Conclusion:**

Our data demonstrate constitutive and regulated expression of the chemokine CX_3_CL1 and its receptor CX_3_CR1 by neurons/astrocytes and microglia, respectively, within the normal and inflamed rat brain. Our findings propose a mechanism by which neurons and reactive astrocytes may control migration and function of the surrounding microglia. In addition, the accumulation of CX_3_CR1 expressing cells other than microglia within the inflammatory brain lesions indicate a possible role for CX_3_CL1 in controlling invasion of peripheral leucocytes to the brain.

## Background

Chemokines are key mediators controlling infiltration of leukocytes to inflamed areas. They consist of a class of related proteins that exert chemotactic properties on leukocytes via interactions with select members of the G-protein coupled, cell membrane-spanning receptors (GPCRs). The so far 50 identified chemokines are divided into 4 subgroups, the XC, CC, CXC and CX_3_C chemokines, and the corresponding GPCRs are accordingly denominated XCR (presently one member), CCR (11 deorphanized members), CXCR (6 members) and CX_3_CR (one single member). Synthesis of chemokines is rapidly induced in damaged or infected tissues and the cell-specific expression of chemokine receptors combined with the situation-specific production of their chemokine ligands provide cues to attract appropriate cell populations to combat invading organisms and neoplastic cells and to clear and repair damaged tissues. Chemokines are also thought to drive chronic inflammatory processes and this have fuelled hopes that pharmacological intervention of ligand-triggered activation of chemokine receptors may serve to reduce clinical manifestations in disorders with inflammatory components [1].

CX_3_CL1 (alternative names: fractalkine or neurotactin) was identified in 1997 as a chemokine of 373 amino acids with, for the chemokine family, an atypical structure, a chemokine domain tethered on top of a mucin-like domain which is followed by a single transmembrane spanning domain and a short cytoplasmic tail [2-4]. CX_3_CL1 is expressed within the brain, heart, lung, kidney, muscle and testis [4-8] where it interacts with a single GPCR, CX_3_CR1 [9,10] to trigger chemotaxis and adhesion [10-13] of CX_3_CR1 expressing cells, including neutrophils, monocytes, NK cells and Th-1 polarized T cells [10,14]. Studies of expression profiles, functional role in *in vitro *and *in vivo *systems and genetic associations to diseases have provided promising clues to a potential role of CX_3_CL1 and its receptor in, among others, rheumatoid arthritis [15-17], allograft rejections [18] and atherosclerosis [19-21].

The physiological role of CX_3_CL1 and its receptor in the brain, however, is less clear. Expression of CX_3_CL1 within the brain is localized to neurons [22-25], whereas CX_3_CR1 is expressed by brain microglia [6,24-26]. Axotomy of the facial nerve trigger increased expression of CX_3_CL1 among the severed motor neurons in the facial nucleus [6] and a similar response was recently observed following intraparenchymal injection of prion proteins [22]. Induction of experimental autoimmune encephalomyelitis (EAE; an animal model for multiple sclerosis) in the mouse has also been associated with CX_3_CL1-like immunoreactivity in blood vessels within the inflammatory brain lesions [3]. This finding is complemented by the increased levels of CX_3_CR1 mRNA in the spinal cord of rats with EAE, as demonstrated by RNAse protection assay [27]. CX_3_CL1 has moreover been shown to regulate microglia functions, including CX_3_CL1-induced mobilization of intracellular Ca^2+^, chemotaxis and the inhibition of Fas-mediated apoptosis *in vitro *[6,23,28]. This is reflected in the activation of microglia following injection of CX_3_CL1 *in vivo *to the rat brain parenchyma [22], indicating a potential role for CX_3_CL1 and CX_3_CR1 in mediating neuronal-microglial cross talk under normal and pathological conditions. In addition, previous claims of CX_3_CL1 expression in endothelial cells [3,15,16,29-32], combined with CX_3_CR1 on monocytes and Th-1 cells [5,14-16,33,34] may indicate a role in attracting pathogenic cells to sites of neuroinflammation, as well.

Multiple sclerosis (MS) is a chronic inflammatory disease of the CNS. It is associated with local activation of microglia and astroglia, infiltration of activated macrophages and T cells, active degradation of myelin and damage to axons and neurons. The disease often develops from isolated self-limiting episodes with various neurological manifestations, including paralysis, to persistent, accentuated loss of neurological functions. The neuroinflammatory component of the disease is, at least in initial phases, thought to mediate important aspects of the clinical manifestations. The proposed role for CX_3_CL1 in the control of microglia activation and leukocyte infiltration places this chemokine/receptor pair in a potentially strategic position to control key aspects in the pathological events that are associated with development of brain lesions in MS. In this study, we examine this hypothesis further by analyzing the distribution, kinetics, regulation and cellular origin of CX_3_CL1 and CX_3_CR1 mRNA expression in the CNS of rats with MOG-induced EAE. The expression of CX_3_CL1 and its receptor was studied with *in situ *hybridization histochemical detection of their mRNA with radio labeled cRNA probes in combination with immunohistochemical staining of phenotypic cell markers. Our findings suggest that CX_3_CL1 and its receptor may control aspects of the neuroinflammatory processes in MOG-induced EAE, and possibly also MS.

## Methods

### Animals

Inbred female DA.RT1^av1 ^rats were obtained from B&K Sollentuna, Stockholm, Sweden. All rats were housed under specific pathogen-free conditions to keep the influence of additional environmental factors, beside immunization, as low as possible. Female DA rats 10–14 weeks of age (150–200 g) were used. All animal experiments were approved and performed in accordance with Swedish national guidelines.

### Preparation of MOG

The N-terminal sequence of rat MOG (amino acids 1–125) was expressed in Escherichia coli and purified to homogeneity by chelate chromatography [35]. The purified proteins in 6 M urea were then dialyzed against PBS to obtain a preparation that was stored at -20°C.

### Induction and assessment of EAE

Rats were anaesthetized with methoxyflurane and injected intradermally at the base of the tail with 0.2 ml inoculum, containing 20 μg recombinant rat MOG in saline, emulsified (1:1) with incomplete Freund's adjuvant (IFA; Difco, Detroit, MI). Rats were clinically scored and weighted daily from day 7 post- immunization (p.i.) until day 30 p.i. by two alternating investigators. The clinical scoring was as follows: 0 = no illness, 1 = tail weakness or tail paralysis, 2 = hind leg paraparesis, 3 = hind leg paralysis, 4 = complete paralysis, moribund state, or death. A disease remission was defined as an improvement in disease score from either 3 or 4 to 1, or from 2, 3 or 4 to 0 that was maintained for at least 2 days consecutively. A relapse was defined as an increase in the clinical deficit of at least two points that lasted for at least 2 days.

### Histopathology

Tissues where obtained from healthy, non-immunized rats or rats sampled on day 8, 13, 18, 21, 24, 29 and 40 p.i. Rats were deeply anaesthetized with methoxyflurane and subjected to perfusion via aorta with 4% paraformaldehyde. Organs were dissected out, routinely embedded in paraffin wax and sectioned at 5 μm. Histopathological evaluation was performed on transverse sections of the forebrain, midbrain, brainstem and 17 different rostro-caudal levels of the spinal cord, using hematoxylin and eosin (HE), Luxol fast blue/-periodic acid Schiff's (PAS) staining and Bielschowsky silver impregnation, to assess inflammation, demyelination, and axonal pathology, respectively [36,37].

### Preparation of radioactively labeled cRNA probes

Preparation of radioactively labeled cRNA probes encoding rat CX_3_CL1 and CX_3_CR1 was carried out as previously described [38]. Briefly, antisense and sense cRNA probes were transcribed in vitro with T3 or T7 RNA polymerase in the presence of ^35^S-uridine triphosphate (^35^S-UTP; NEN – DuMedical, Sollentuna, Sweden). After removal of unincorporated nucleotides by Quick Spin columns (Boehringer -Mannheim, Indianapolis, IN), the specific activities of all the probes were 1–3 × 10^9 ^dpm/ug. The CX_3_CL1 and CX_3_CR1 cRNA probes were transcribed from cDNA fragments cloned into pDP18 CU minus plasmid vector (Ambion, Austin, Texas). These cDNA fragments correspond to a 450 base pair cDNA fragment of rat CX_3_CL1 (from 20–469 bp, GeneBank accession number AF030358) and a 882 base pair cDNA fragment of rat CX_3_CR1 (GenBank accession number RN04808), respectively, and were generated by RT-PCR using sequence-specific oligonucleotide primers. The identities of the cloned cDNA fragments were finally confirmed by sequencing and database comparisons. Restriction enzymes and RNA polymerases were obtained from Promega (Madison, WI).

### In situ hybridization and immunohistohistochemistry

To detect expression of CX_3_CL1 and CX_3_CR1 mRNA, *in situ *hybridization experiments were performed on sections from rat CNS. Hybridization and autoradiography were carried out as previously described [39]. Briefly, tissue sections were mounted on Superfrost plus slides (Super Frost Plus, Pittsburgh, USA) and dried under vacuum overnight after deparaffination in xylene, pre-treated in a microwave oven for 10 minutes at 97°C in 10 mM SSC (pH 6.0) and dehydrated in ethanol. As controls, radio labeled sense probes were transcribed in the sense orientation and hybridized to slides as processed in parallel. After application of 100 ul of hybridization solution containing 10^6 ^cpm of the cRNA probes, the slides were cover slipped and incubated at 60°C for 16 to 20 hours. Slides were subsequently washed in 4× standard saline citrate (SSC, pH 7.0), digested in 20 υg/ml ribonuclease A solution at 37°C for 30 minutes, washed in decreasing concentrations of SSC, ending with 0.1 × SSC for 30 minutes at 70°C.

To identify the cellular phenotypes of the CX_3_CL1 or CX_3_CR1 expressing cells, an immunohistochemical staining protocol was directly applied following the *in situ *hybridization step, as previously described [39]. The following monoclonal primary antibodies were used: an antibody specific for rat monocytes and macrophages (ED-1, Serotec, diluted 1/500) or an antibody reactive with glial fibrillary acidic protein (GFAP; G-A-5 diluted 1/20; Boehringer – Mannheim). Analysis for expression of CX_3_CL1 or CX_3_CR1 in neurons was performed, using an antibody reactive to a neuronal specific protein (NeuN [40]) diluted 1/100, Chemicon). Notably, as the NeuN antigen proved to be sensitive to the present conditions for combined labeling of mRNA and protein, we selected to compare the distribution of NeuN protein and CX_3_CL1 or CX_3_CR1 mRNA independently on consecutive tissue sections. A biotinylated sheep anti-mouse IgG antibody (Life Sciences) served as the secondary reagent, with the avidin biotin peroxidase (ABC) detection system (ABC Elite, Vector Laboratories). Finally, a biotinylated lectin (GSI-B4, Vector Laboratories) combined with the ABC detection system was used for the detection of vascular endothelial cells and macrophages and microglia in various stages of activation. Parallel tissue sections were incubated without primary antibody as control of specificity of the staining.

### Quantification of CX_3_CR1 mRNA-expressing cells in defined lesional stages

In a total of 5 brain sections from 4 rats in the relapse stage (days 21–45), 10 inflammatory lesions were selected and defined according to the stage of demyelinating activity as previously described [41]. Briefly, early active (EA) lesions were characterized by dense infiltrates of macrophages, lymphocytes and microglia. Myelin sheaths were in the process of disintegration and macrophages contained luxol fast blue (LFB)-stained myelin degradation products. Late active (LA) lesions were still densely populated by macrophages. Damaged myelin had been removed from the axons and macrophages contained PAS-positive myelin degradation products. Inactive completely demyelinated (DM) lesions showed no evidence of ongoing tissue destruction. Inflammatory cells were present, but macrophages did not display LFB or PAS staining. A single plaque usually contained two or more different stages of lesional activity (e.g. a central DM core surrounded by LA and EA areas). The region in the immediate vicinity of the plaques, showing no microscopic signs of demyelination, was defined as periplaque white matter (PPWM). Representative regions outside lesions and PPWM areas were defined as normal white matter (NWM) and served as internal controls. Following *in situ *hybridization and GSI-B4 isolectin immunohistochemistry, the brain sections (3 from the cerebellum and pons, 1 from the frontal cortex, 1 from the thalamus) were captured with a Kappa DX-20 digital camera mounted on a Nikon E600 microscope. In each of the defined lesions, the total number of GSI-B4+ cells as well as the numbers of GSI-B4+ and GSI-B4- cells with positive hybridization signals for CX_3_CR1 were counted in a standardized field of 1.9 × 10^4 ^μm^2^. The intensity of the in situ hybridization signal was determined by counting areas of silver grains exceeding the mean background level (as measured over 30 similarly sized areas outside the cellular borders) + 11 SD (Access Analysis system, Euromed Networks). In total, 8 EA lesions, 8 LA lesions, 7 DM lesions, 8 PPWM areas and 9 NWM areas were included in the analysis. Data were statistically evaluated with ANOVA, followed by Tukey\primes multiple comparison test. A *P *value < 0.05 was considered to be statistically significant.

### Imaging

Bright-field images were captured with a Kappa DX-30 digital camera mounted on a Leica microscope. Digital images were imported into Adobe Photoshop (v. 6.0), where they were adjusted to balance and optimize brightness, contrast, and sharpness. Individual files were exported to Canvas (v. 8.0) for assembly into plates, which were rendered at initial resolution of 300 dpi.

## Results

### Expression of mRNA encoding CX_3_CL1 and its receptor in the normal rat brain and spinal cord

**Figure 1:**
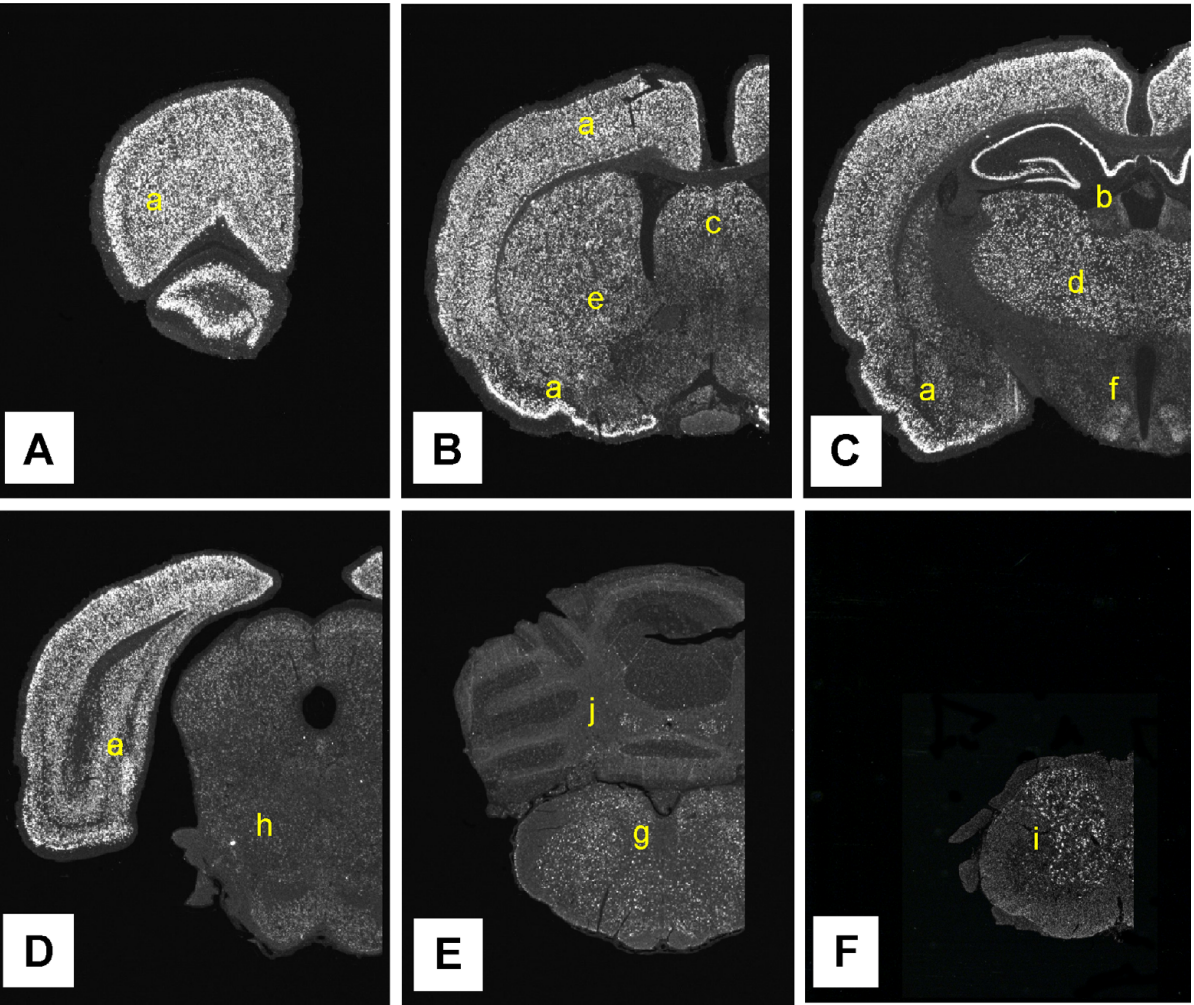
**Distribution of CX_3_CL1 mRNA expressing cells in the normal rat brain**. Coronal sections sampled at regular intervals throughout the rostro-caudal extent of the normal rat brain hybridized with a 35S-labeled antisense-CRNA probe encoding rat CX_3_CL1. Cells expressing mRNA encoding the chemokine are visualized as accumulations of white silver grain in this microscopic darkfield illumination at low magnification. The letters in each subfigure refer to the approximate levels according to the Paxinos stereotaxic brain atlas [61]. The highest levels of CX_3_CL1 mRNA were detected exclusively within the grey matter of the cerebral cortex (A:a, B:a, C:a, D:a), hippocampus (C:b), septum (B:c), thalamus (C:d) and striatum (B:e). Medium to low level expression was detected in the hypothalamus (C:f), pons (E:g), mesencephalon (D:h), medulla oblongata (not shown) and spinal cord (F:i). The cerebellum (E:j) was devoid of CX_3_CL1 expression, except for a low level of expression in the deep cerebellar nuclei. Parallel sections hybridized with a sense-transcribed CX_3_CL1 cRNA probe of equal specific activity did not reveal signals above background levels.

**Figure 5:**
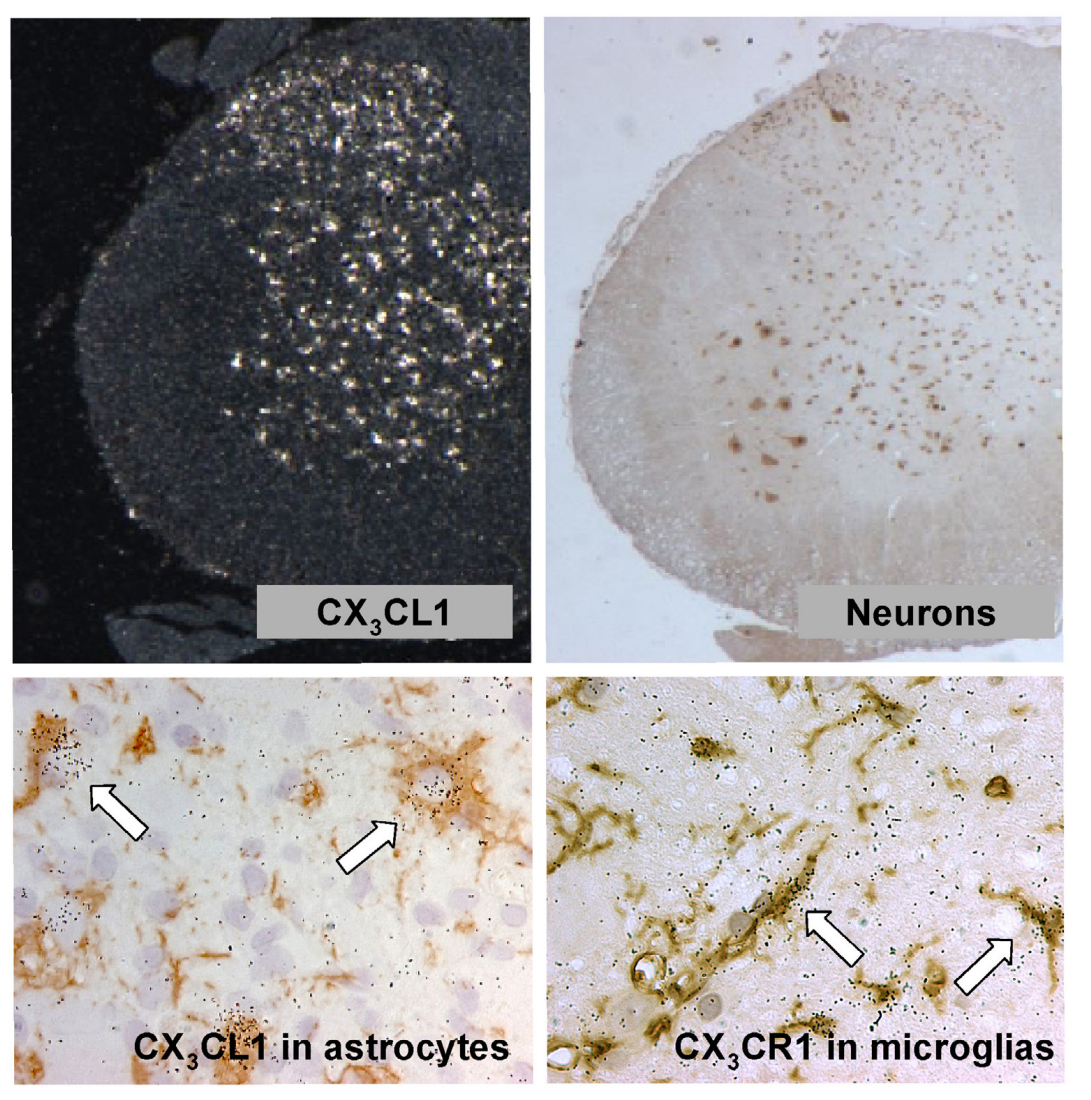
**Phenotyping of CX_3_CL1 and CX_3_CR1 expressing cells**. CX_3_CL1 mRNA expressing cells (upper left). Immunohistochemical staining for the neuronal marker NeuN (upper right) performed on a parallel tissue section. Dual staining for CX_3_CL1 mRNA and the astrocyte marker GFAP (lower left). Combined staining for CX_3_CR1 mRNA and the microglial marker GSI-B4 isolectin (lower right).

Radio labeled antisense cRNA probes transcribed from cDNA encoding rat CX_3_CL1 (Fig. 1) and its receptor, CX_3_CR1 (data not shown), were initially hybridized *in situ *to 5 μm tissue section obtained throughout the entire rat brain and spinal cord. In the forebrain, cells expressing high levels of CX_3_CL1 mRNA were detected throughout the olfactory bulb, cerebral cortex, amygdala, globus-pallidus and thalamus (Fig. 1). These cells manifested, with few exceptions, a neuronal phenotype and co-distributed with cells staining positive for the pan-neuronal marker NeuN (exemplified in Fig. 5a, b at the spinal cord level). High levels of CX_3_CL1 mRNA expression were also detected in all pyramidal cells within the hippocampal formation (Fig. 1). Significantly lower, but still clearly detectable, levels of CX_3_CL1 mRNA were observed within all aspects of the hypothalamus (Fig 1), with neurons within the ventromedial nucleus displaying the strongest labeling. In the mesencephalon, pons, medulla oblongata and the spinal cord low to medium expression levels were uniformly detected in neuronal-like cells. A notable exception was the cerebellum, where very low levels of expression were detected within granule and pyramidal cells whereas neuronal like cells in the deep cerebellar nuclei expressed CX_3_CL1 mRNA at medium levels (Fig. 1). Tissue sections hybridized in parallel with a radio labeled cRNA probe transcribed in the sense orientation did not reveal any hybridization signal above background levels (data not shown).

In addition to the expression of CX_3_CL1 mRNA among neuronal-like cells within the rat brain and spinal cord, we also detected low-to medium expression of CX_3_CL1 in a few, solitary cells dispersed throughout the white matter areas. These cells manifested a flattened, elongated morphology (data not shown) and were not positively stained in dual labeling experiments for CX_3_CL1 mRNA with phenotypic markers for macrophages/microglias or astrocytes. No expression of CX_3_CL1 mRNA was detected in cells associated with the cerebral vasculature and the meninges, including the endothelial cells themselves.

In consecutive tissue sections, *in situ *hybridization with a radio labeled antisense cRNA probe encoding rat CX_3_CR1 revealed a low-to-medium expression within cells uniformly distributed throughout the entire neuraxis (data not shown). The morphology of these cells, as well as their positive labeling with GSI-B4 (*Griffornia simplifolica *isolectin B4; stains microglia, macrophages and endothelial cells) identified them as being inactive microglia (data not shown). No detectable expression was observed over other cells, including neurons and perivascular and meningeal macrophages. Hybridization with a sense cRNA probe transcribed from the same cDNA did not reveal a signal above background levels (data not shown).

### Expression of mRNA encoding CX_3_CL1 and its receptor in the CNS of rats with MOG-EAE

**Figure 2:**
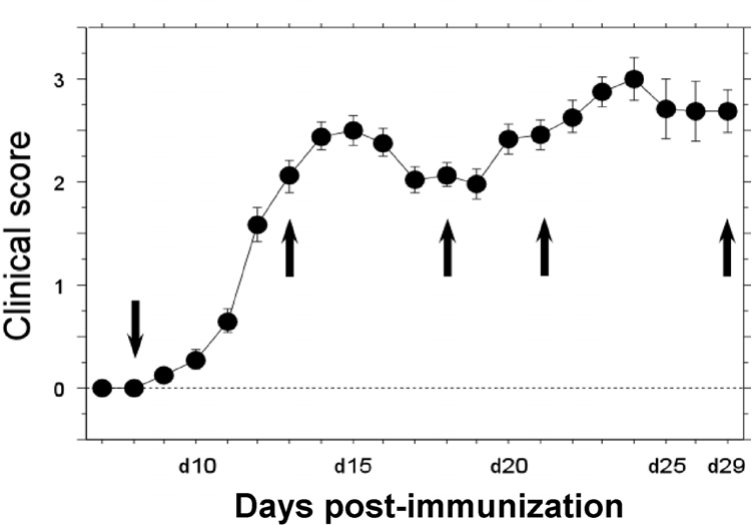
**Sampling of rats from various clinical stages of MOG-EAE**. Female DA rats (n = 20) were immunized with mineral oil-emulsified MOG and evaluated daily for severity of paralysis. The arrows indicate selected time point at which subsequent kinetic analyses were performed. Rats (n = ≥ 3/group) which conformed in the clinical score curve above were perfused transcardially, tissues were dissected out and subjected to histopathological analysis of encephalitis and distribution of CX_3_CL1 and CX_3_CR1 expressing cells.

**Figure 4:**
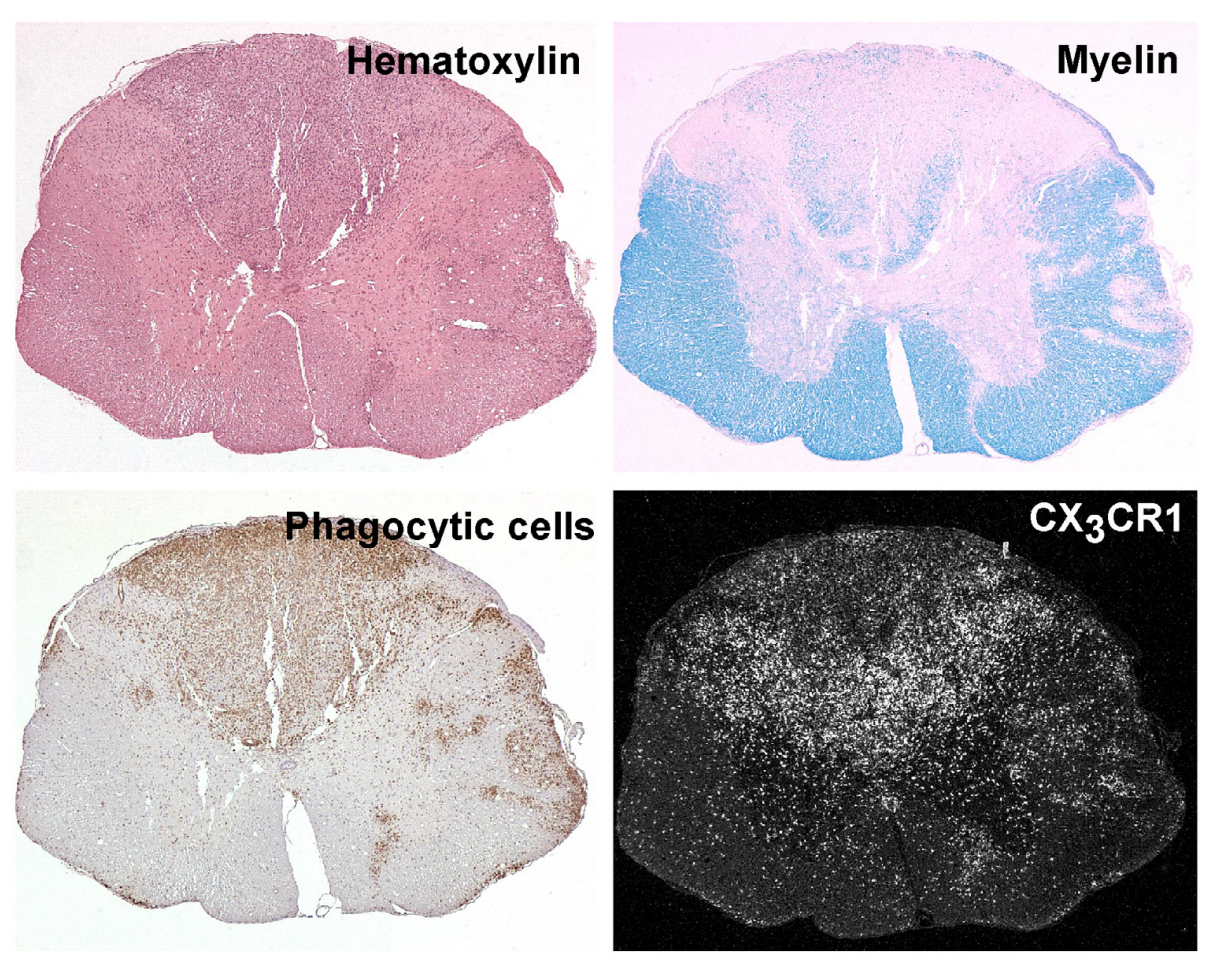
**Sublesional distribution of CX_3_CR1 expressing cells**. Cellular infiltrations in the MOG-EAE rat brain (early relapse phase; day 21 post immunization) are observed with hematoxylin-eosin staining (upper left). Areas of active demyelination are revealed with Luxol fast blue/PAS staining (upper right). Actively phagocytozing cells are detected with immunohistochemical staining for ED-1 (lower left). This figure exemplifies the uneven distribution of CX_3_CR1 mRNA expressing cells (lower right) within the inflammatory aggregates.

To explore the role of CX_3_CL1 and its receptor in the control of inflammatory cascades in MS, we examined their expression and regulation in the CNS of rats with MOG-induced EAE. In inbred DA rats, this disease manifested a mostly relapsing-remitting disease course with an initial paralytic episode, commencing around day 9–13 followed by a partial or complete remission and then a relapse of paresis (Fig. 2). In some rats the initial (acute) paresis progressed directly into a prolonged paralysis without any intervening remission of symptoms. In a minority of the rats the acute episode spontaneously resolved without further clinical signs of disease. The acute phase of paresis was characterized histopathologically by astroglial and microglial activation and perivascular and submeningeal infiltration of lymphocytes, macrophages and granulocytes. The inflammatory lesions were mostly confined to the spinal cord and, in some rats, the optic nerve and the cerebellar white matter. In rats that exhibited a clinical relapse the inflammation followed generally the pattern observed during the acute phase with submeningeal and perivascular lesions. However, the inflammatory reaction was often more extensive with numerous confluent lesions that covered substantial areas of the spinal white matter (Fig. 4), sometimes extending into the grey matter areas. Inflammatory lesions were at this stage closely associated with a marked demyelination as well as axonal degeneration and loss of axonal density. There was a distinct transition in cellular composition observed within the inflammatory lesions, from predominantly lymphocytes and granulocytes in the acute phase lesions to an overwhelming presence of macrophages and microglia with only a few granulocytes throughout the inflammatory lesions during the clinical relapse. These phagocytic cells exhibited obvious signs of uptake and degradation of myelin components. This pattern of paresis and histopathological alterations was generally in accordance with previous studies [37,42], to which interested readers are referred for further details on the disease pathology.

For the following studies we selected rats that conformed to the typical relapsing-remitting disease phenotype (Fig. 2). *In situ *hybridization experiments revealed an uncompromised neuronal expression of CX_3_CL1 mRNA throughout the entire CNS at all time points examined, except for a tendency to a reduced level of expression in grey matter regions which were notably infiltrated with inflammatory cells (data not shown). In addition, increased expression of CX_3_CL1 mRNA was evident in a small number of non-neuronal cells within the inflammatory lesions. Double-labeling experiments showed those cells to stain positive for the astroglial marker glial fibrillary acidic protein, GFAP (Fig. 5). No hybridization signal above background levels was detected in cells staining positive for GSI-B4 (e.g. macrophages/microglia and endothelial cells). Tissue sections hybridized in parallel with a sense-transcribed CX_3_CL1 cRNA probes did not generate hybridization signals above background levels (data not shown).

**Figure 3:**
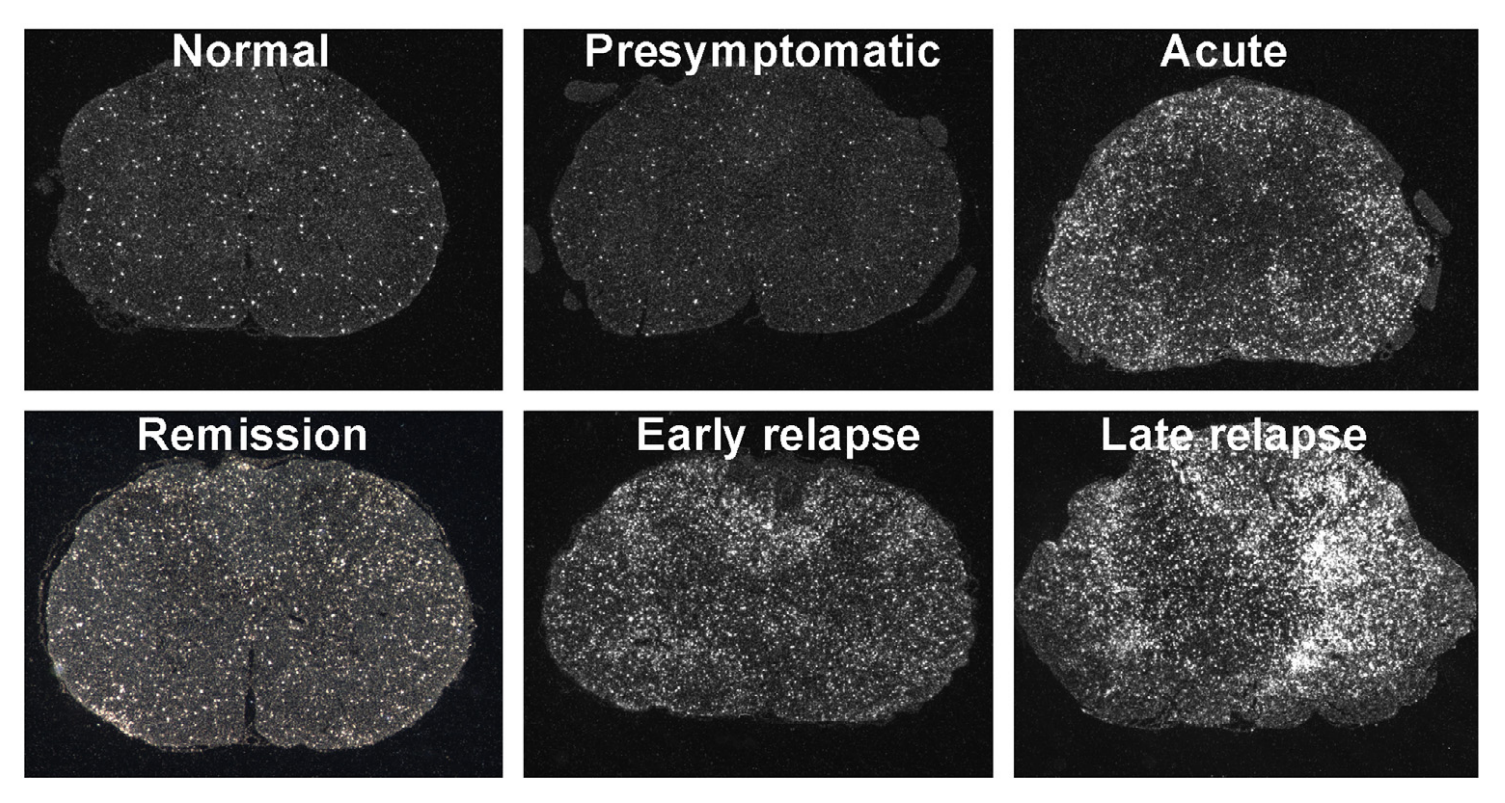
**Distribution of CX_3_CR1 mRNA expressing cells in spinal cord of EAE rats**. *In situ *hybridization with a radiolabeled antisense cRNA probe encoding rat CX_3_CR1 to coronal sections from the lumbar segment of spinal cord of rats with MOG-EAE. Cells expressing CX_3_CR1 mRNA are visualized by darkfield illumination of the photoemulsion-dipped slides.

*In situ *hybridization with an antisense cRNA probe for CX_3_CR1 demonstrated a low, constitutive expression of the receptor in cells evenly distributed throughout the spinal cord of healthy, non-immunized control rats (n = 3) and presymptomatic rats (day 8 p.i, n = 3) (Fig. 3). The morphology and positive staining for GSI-B4 identified these cells as being inactive microglial cells. Rats examined at various stages following onset of clinically manifest MOG-induced EAE (day 13 p.i, acute phase, n = 3; day 18 p.i, remission phase, n = 3; day 21 p.i, early relapse phase, n = 3; day 24 p.i, mid relapse phase, n = 3; day 29 p.i, late relapse phase, n = 3) demonstrated a clear visual increase in CX_3_CR1 mRNA levels per cell, as well as a notably increased density of CX_3_CR1 mRNA expressing cells, within the inflammatory areas (Fig. 3 and 4). These aggregates of CX_3_CR1 mRNA expressing cells were closely overlapping with the inflammatory lesions, being prominent in the perivascular and submeningeal lesions during the acute phase and with a few remaining lesions detectable during the remission phase. In rats examined at various time points of the relapse phase the aggregation of cells expressing high levels of CX_3_CR1 mRNA closely followed the areas of expanding lesions. The great majority of the CX_3_CR1 mRNA expressing cells were at all stages of disease positively stained with GSI-B4 isolectin as well as with the marker for active phagocytosis, ED-1. While all GSI-B4 positive, CX_3_CR1 mRNA expressing cells in PPWM regions displayed abundant ramified processes identifying them as resident microglia, the morphology of the GSI-B4 positive, CX_3_CR1 mRNA expressing cells within EA, LA and DM lesions was consistent with both macrophages and microglia in an activated, phagocytic state (Fig. 5). Occasional GSI-B4 negative, CX_3_CR1 mRNA expressing cells were identified within the inflammatory lesions. These cells had rounded or slightly elongated nuclei and were of lymphocyte-like size. However, the great majority of lymphocytes within the lesions did not express CX_3_CR1mRNA (data not shown).

### Quantification of CX_3_CR1 mRNA-expressing cells in relation to the stage of demyelinating activity

**Figure 6:**
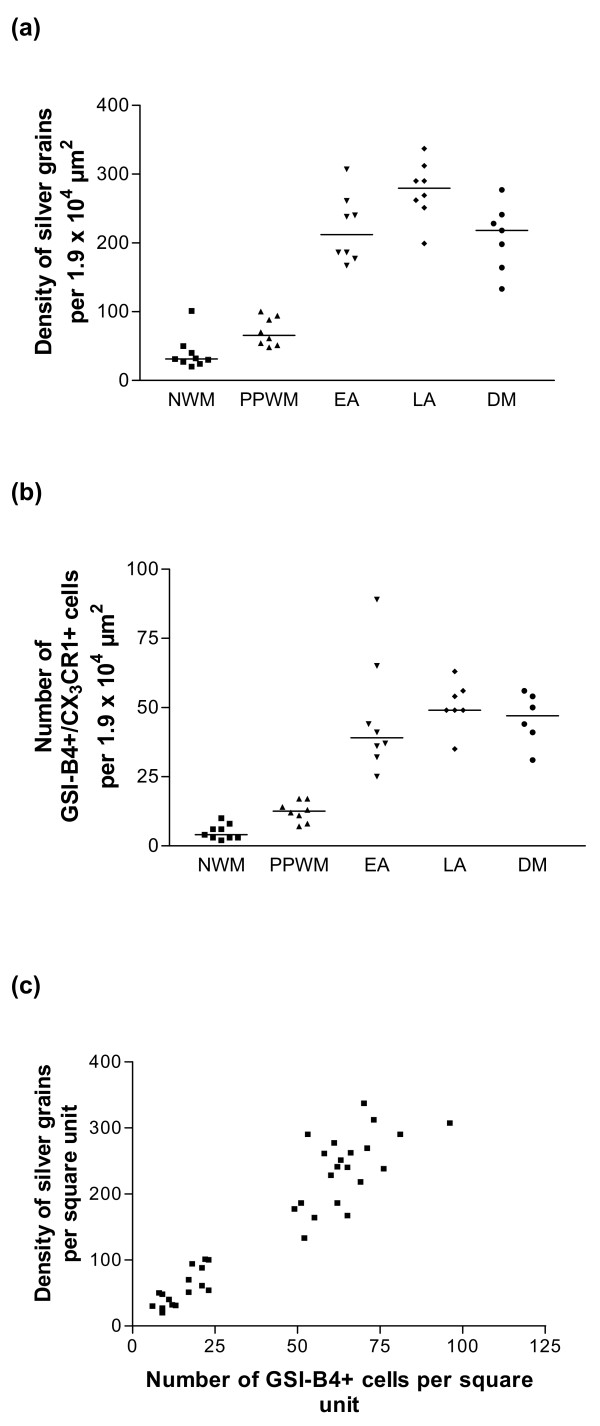
**Quantification of CX_3_CR1 mRNA expressing cells in defined lesional stages**. (a) Density of silver grains (defined as areas exceeding the mean background + 11 SD) per square unit. (b) Number of CX_3_CR1 mRNA-expressing cells positively stained with GSI-B4 isolectin. (c) Correlation between total number of GSI-B4 cells and intensity of in situ hybridization signal (density of silver grains per square unit).

To further characterize the distribution of CX_3_CR1 mRNA expressing cells in MOG-induced EAE, we performed a quantitative analysis in defined time-staged lesions. The strongest expression of CX_3_CR1 mRNA was found in LA areas, which contained a significantly higher density of silver grains per square unit compared to EA areas (*p *< 0.05), DM areas (*p *< 0.05), PPWM regions (*p *< 0.001) and NWM (*p *< 0.001) (Table 1, Figure 6a). In EA and DM areas, the expression of CX_3_CR1 mRNA was significantly higher compared to PPWM (*p *< 0.001) and NWM (*p *< 0.001). Although PPWM regions tended to show a higher density of silver grains per square unit compared to NWM, this difference was not statistically significant (Table 1, Figure 6a). The actual number of CX_3_CR1 mRNA expressing cells was three-fold higher in lesional areas compared to PPWM and NWM but did not differ significantly between EA, LA and DM. The overwhelming majority of the CX_3_CR1 mRNA expressing cells stained positive for GSI-B4 isolectin, identifying them as macrophages/microglia (Table 1, Figure 6b). Although not all GSI-B4+ cells expressed CX_3_CR1 mRNA, there was a significant (*p *< 0.0001) correlation between the total number of GSI-B4+ cells and the density of silver grains per square unit (Table 1, Figure 6c).

**Table 1:** Quantification of CX_3_CR1 mRNA expressing cells per square unit in rat EAE lesions (mean ± SEM)

	**NWM**	**PPWM**	**EA**	**LA**	**DM**
Density of silver grains* per 1.9 × 10^4 ^μm^2^	39.4 ± 8.2	70.8 ± 7.2	220.3 ± 17.4^a^	276.3 ± 14.8^b^	208.4 ± 18.3^a^
**Total number of GSI-B4+ cells per 1.9 × 10^4 ^μm^2^**	11.0 ± 1.5	18.6 ± 1.6	65.3 ± 5.3^a^	68.1 ± 3.3^a^	59.8 ± 2.4^a^
**Number of GSI-B4+/CX_3_CR1+ cells per 1.9 × 10^4 ^μm^2^**	5 ± 0.9	12.4 ± 1.3	46.1 ± 7.4^a^	50.7 ± 3.3^a^	46.0 ± 3.8^a^
**Number of GSI-B4-/CX_3_CR1+ cells per 1.9 × 10^4 ^μm^2^**	0 ± 0	0 ± 0	0.8 ± 0.3	2.1 ± 0.7^a^	0.8 ± 0.4

*Silver grains: defined as areas exceeding the mean background level + 11 SD

^a ^Statistically significant against NWM and PPWM

^b^Statistically significant against NWM, PPWM, EA and DM

## Discussion

This study was designed to provide further insights into the mechanisms of proinflammatory cell trafficking into the CNS in MS. Our results show that the chemokine CX_3_CL1 is expressed, and actively regulated, within inflammatory CNS lesions in rats with MOG-induced EAE, a rodent model of MS. In addition, we demonstrate that cells expressing the CX_3_CR1 receptor, the majority of which are potentially pathogenic phagocytic cells (i.e. macrophages and/or microglia), are densely accumulating within the inflammatory lesions. This is consistent with the hypothesis for a role of CX_3_CL1-CX_3_CR1 in the local control of leukocyte infiltration into CNS lesions in MOG-EAE rats, and possibly also MS. The constitutive CX_3_CL1 and CX_3_CR1 expression by neurons and microglia, respectively, even in the healthy rat brain, indicates a potential mechanism whereby neurons control microglia functions in the intact brain tissue, as well.

Our present findings of constitutive expression of CX_3_CL1 within the normal rat brain are in agreement with previous reports where CX_3_CL1 mRNA [24,25,43] and protein [25,43] was demonstrated in most CNS neurons of healthy rats. Similar results have also been obtained in the mouse [4], rhesus monkey [25] and human [44] brain. It is interesting to note that the levels of CX_3_CL1 mRNA differed greatly between different CNS regions, possibly indicating a more pronounced role for neuronal CX_3_CL1 in many forebrain structures, i.e. cerebral cortex, hippocampus and striatum. Our demonstration that CX_3_CL1 mRNA expression is induced in astrocytes within EAE lesions is novel although the data are in accordance with study of humans with HIV-associated dementia where CX_3_CL1 immunoreactivity was detected in astrocytes [45]. Induced expression of CX_3_CL1 in astrocytes was also observed following intraparenchymal administration of prion protein to the rat brain [22]. Co- cultivation of human astrocytes and HIV-infected macrophages also resulted in increased CX_3_CL1 immunoreactivity in astrocytes [45] and stimulation of rat [23] and human [46] astrocytes with TNF-α and IL-1β or INF-γ *in vitro *has been shown to up regulate CX_3_CL1 mRNA levels. A recent immunohistochemical study of MS- and normal brain tissue demonstrates a constituent CX_3_CL1 expression in astrocytes and an increased expression in human adult astrocyte cultures stimulated with pro-inflammatory cytokines, but fail to demonstrate an upregulation of CX_3_CL1 in MS patients [47]. However, these data collectively indicate that astrocytes are capable of upregulating CX_3_CL1 expression in inflamed or injured brain tissues.

In contrast to the observed expression of CX_3_CL1 in neurons and astrocytes, we were not able to confirm previous findings in EAE-afflicted mice [2] where endothelial immunoreactivity for CX_3_CL1 was demonstrated within inflamed brain lesions. Our findings are corroborated by a recent study by Schwaeble et al. [25] in which no CX_3_CL1 mRNA expression was detected within the endothelium of EAE rats and mice. Possible explanations for the contradictory results are differences in the sensitivity of the detection methods used or a potential non-specific, immunohistochemical cross-reactivity to other proteins within the endothelium in the study by Bazan et al. [2]. An alternative explanation would be if the secreted form of CX_3_CL1, which is produced within the inflamed brain parenchyma by neurons and astrocytes, is transcytosed in an abluminal-to-luminal direction for presentation to blood leukocytes in a similar manner to what previously have been described for IL-8 and RANTES [48]. It is, however, important to note that endothelial cells outside the CNS have convincingly been demonstrated to express CX_3_CL1 mRNA and protein [6,8,29-32]. CX_3_CL1 expression by the brain endothelium, which may be below the detection limit of our assay, therefore remains a tantalizing possibility by which blood leukocytes may become attracted to migrate across the blood-brain barrier into inflammatory CNS lesions of MOG EAE rats.

Our present studies of CX_3_CR1 expression in the rat brain confirm previous findings by Harrison et al [43], Nishiyori et al [24] and others by demonstrating that microglia express CX_3_CR1 mRNA constitutively within the normal rat brain. These findings are also consistent with previous demonstrations that CX_3_CL increases microglial migration [43], proliferation [49], survival [28], intracellular recruitment of calcium [43] and secretion of cytokines and metalloproteases [50]. In addition, we demonstrate that CX_3_CR1 mRNA expressing cells are rapidly accumulating at high densities within the inflammatory CNS lesions of rats with MOG-induced EAE, confirming previous observations [41]. Interestingly, those cells amassed throughout most kinetic stages of the lesions, with the most conspicuous densities detected in the late active stages. It is tempting to speculate about a down-regulatory role of CX_3_CR1 expression in these late lesional stages, based on the possible induction of nonsignaling CX_3_CR1 receptors [51]. The overwhelming majority of the CX_3_CR1 mRNA expressing cells within the lesional areas were phagocytic cells (i.e. macrophages and/or microglias) as demonstrated in double-labeling protocols with the GSI-B4 isolectin or the ED-1 antibody. However, a few additional, non-GSI B4 labeled, CX_3_CR1 mRNA expressing cells were also detected within the CNS lesions. These cells may be speculated to correspond to infiltrating blood leukocytes, such as NK cells, γδ T cells and/or terminally differentiated CD4^+ ^and CD8^+ ^T cells, which previously have been described to express CX_3_CL1 receptors and/or to respond functionally upon CX_3_CL1 stimulation [14,15,33,52,53]. However, the majority of the lymphocyte-like cells within the inflammatory lesions of the present rats did not express CX_3_CR1 mRNA. Notably, no expression of CX_3_CR1 mRNA was detected in GFAP-labeled astrocytes (data not shown) or neuronal-like cells. This contrasts to earlier findings of CX_3_CR1-like immunoreactivity within neurons of a rat model for prion disease [22], in humans with HIV encephalitis [44] and in vitro cultivated hippocampal neurons [54]. Hulshof et al. [47] found weak to moderate neuronal CX_3_CR1-like immunoreactivity in the cortical grey matter, depending on the tissue sample observed. Those data suggest that some populations of neurons may indeed have the capacity to express CX_3_CR1 under certain conditions.

Our present data are thus consistent with a role for CX_3_CL1 in the rat CNS, both during healthy and inflammatory conditions. The constitutive expression of CX_3_CL1 and its receptor on neurons and microglia respectively demonstrates that this chemokine-receptor pair is not normally promoting inflammation. Their function may under these conditions possibly be inert but CX_3_CL1, which is normally linked physically to the neuronal membrane via a spacer domain and a transmembrane spanning motif [2], may alternatively mediate a direct contact between neurons and neighboring microglia via its proadhesive properties [2]. Microglia are highly reactive to insults to brain tissue and may inflict permanent damage if not kept in check. A CX_3_CL1-mediated interaction between neurons and surrounding microglias may under healthy conditions provide a mechanism by which neurons subdue the proinflammatory and neurotoxic capacity of microglia. This notion is supported by recent findings from in vitro studies where CX_3_CL1 inhibited neuronal death following the stimulation in vitro of cocultured microglia and hippocampal neurons with lipopolysaccharide, LPS [55]. In this study, CX_3_CL1 was also demonstrated to inhibit LPS stimulated activation of microglia and associated TNF-α synthesis [55]. Moreover, endogenous CX_3_CL1 has also been shown to inhibit the increased levels of TNF-α and 8-isoprostane in the hippocampus and cerebrospinal fluid following intracerebroventricular injections of LPS [56]. In another study, CX_3_CL1 protected neurons in vitro from the neurotoxic properties of platelet activating factor or the HIV envelope protein, Tat [44]. In contrast, under conditions where neurons and their axonal and dendritic projections are injured, as is frequently the case in EAE and MS, CX_3_CL1 is likely to be cleaved and released from the neuronal membrane by locally acting metalloproteases [57]. This would create a chemotactic gradient that, in combination with other locally active proinflammatory mediators, may subserve the extensive accumulation and activation of microglia within injured brain sites as observed in the present study. In addition, the soluble form of CX_3_CL1 may also contribute to the infiltration of CX_3_CR1-expressing leukocytes, including macrophages and effector T and NK cells. These cell types have crucial roles in EAE as mediators of tissue destruction and/or disease regulation [58]. A functional significance of the soluble CX_3_CL1 / CX_3_CR1-pathway is suggested by the present demonstration of CX_3_CR1 expression in macrophage-like cells and a small fraction of lymphocyte like cells. Interestingly, concurrent studies have provided functional evidences that CX_3_CR1 and its ligand serve as important mediators of inflammation and pathology in animal models models for atherosclerosis, transplant rejections, glomerulonephritis and stroke [12,19,29,59]. Studies in mice with targeted deletions of the CX_3_CL1 or CX_3_CR1 genes have, however, failed to provide information regarding a contributory function for this chemokine-receptor pair in EAE. CX_3_CL1 ^-/- ^mice [60] or CX_3_CR1 ^-/- ^mice [18] developed EAE in a manner not significantly deviating from wildtype mice. Furthermore, CX_3_CR1 ^-/- ^mice did not manifest alterations in the microglia responses around injured/dying motor neurons in a peripheral axotomy model [53]. These studies suggest a redundant compensatory mechanism by other chemotactic factors or a more subtle role of this chemokine-receptor pair in these mouse models for MS. The aforementioned studies, which proposed a neuroprotective role for CX_3_CL1, would also warrant a more thorough investigation of the long-term outcome as regards paralysis and neuronal injuries in EAE-challenged CX_3_CL1, or CX_3_CR1, deficient mice. A definite assignment of the role of CX_3_CL1 and its receptor in normal and inflamed or injured CNS conditions will await conceptual testing in animal disease models using more informative tools such as selective immunoneutralizing antisera, peptide antagonists or non-peptidergic CX_3_CR1 antagonists delivered at various stages of the disease.

## Conclusion

• We have provided data to demonstrate constitutive and regulated expression of the chemokine CX_3_CL1 and its receptor CX_3_CR1 by neurons/astrocytes and microglia, respectively, within the normal and inflamed rat brain.

• Our findings propose a mechanism by which neurons and reactive astrocytes may control migration and function of the surrounding microglia.

• In addition, the accumulation of CX_3_CR1 expressing cells other than microglia within the inflammatory brain lesions indicate a possible role for CX_3_CL1 in controlling invasion of peripheral leucocytes to the brain.

## Competing interests

The author(s) declare that they have no competing interests.

## Authors' contributions

Design of studies (DS, SE, EW, TO, HL, AE-D), cloning of plasmid construct (AE-D), experimental induction of EAE and preparation of tissues (SE, DS, MN), in situ hybridization and immunohistochemical histochemical stainings (DS, MN), analysis of data (DS, SE, HL, A-LB, AED), writing/reviewing of manuscript (all authors). All authors have read and approved the final manuscript.
